# Supplementary material for: Modeling glioblastoma heterogeneity as a dynamic network of cell states
Source: Mol Syst Biol. 2021 Sep 16;17(9):e10105. doi: 10.15252/msb.202010105 (PMC8444284; doi:10.15252/msb.202010105)
Supplement: Supplementary file 5 — Source Data for Figure 3 [file MSB-17-e10105-s001.zip › Figure3A_sourcedata/GSEA_3065/nosigdb_state3.GseaPreranked.1622718426028/SEURAT_CELLCYCLE.html]

Details for gene set SEURAT\_CELLCYCLE[GSEA]

|  || Dataset | state3 |
| Phenotype | NoPhenotypeAvailable |
| Upregulated in class | na\_pos |
| GeneSet | SEURAT\_CELLCYCLE |
| Enrichment Score (ES) | 0.5765855 |
| Normalized Enrichment Score (NES) | 2.2409804 |
| Nominal p-value | 0.0 |
| FDR q-value | 0.0 |
| FWER p-Value | 0.0 |
Table: GSEA Results Summary

  

Fig 1: Enrichment plot: SEURAT\_CELLCYCLE      
 Profile of the Running ES Score & Positions of GeneSet Members on the Rank Ordered List

  

| PROBE | GENE SYMBOL | GENE\_TITLE | RANK IN GENE LIST | RANK METRIC SCORE | RUNNING ES | CORE ENRICHMENT || 1 | CENPF |  |  | 4 | 0.619 | 0.0584 | Yes |
| 2 | TOP2A |  |  | 42 | 0.448 | 0.0969 | Yes |
| 3 | MKI67 |  |  | 59 | 0.407 | 0.1337 | Yes |
| 4 | NASP |  |  | 85 | 0.360 | 0.1651 | Yes |
| 5 | CENPE |  |  | 126 | 0.321 | 0.1910 | Yes |
| 6 | LBR |  |  | 131 | 0.319 | 0.2209 | Yes |
| 7 | DLGAP5 |  |  | 207 | 0.279 | 0.2388 | Yes |
| 8 | HELLS |  |  | 218 | 0.275 | 0.2638 | Yes |
| 9 | SMC4 |  |  | 265 | 0.255 | 0.2828 | Yes |
| 10 | ANLN |  |  | 281 | 0.249 | 0.3048 | Yes |
| 11 | ATAD2 |  |  | 282 | 0.249 | 0.3285 | Yes |
| 12 | CKAP5 |  |  | 366 | 0.227 | 0.3405 | Yes |
| 13 | KIF20B |  |  | 411 | 0.216 | 0.3560 | Yes |
| 14 | TMPO |  |  | 420 | 0.214 | 0.3755 | Yes |
| 15 | KIF11 |  |  | 451 | 0.209 | 0.3919 | Yes |
| 16 | HMMR |  |  | 478 | 0.202 | 0.4081 | Yes |
| 17 | MCM4 |  |  | 482 | 0.201 | 0.4269 | Yes |
| 18 | ECT2 |  |  | 527 | 0.195 | 0.4404 | Yes |
| 19 | RRM1 |  |  | 537 | 0.194 | 0.4578 | Yes |
| 20 | TPX2 |  |  | 544 | 0.193 | 0.4755 | Yes |
| 21 | KIF23 |  |  | 662 | 0.177 | 0.4788 | Yes |
| 22 | NUF2 |  |  | 680 | 0.174 | 0.4934 | Yes |
| 23 | BUB1 |  |  | 717 | 0.168 | 0.5052 | Yes |
| 24 | CASP8AP2 |  |  | 794 | 0.158 | 0.5115 | Yes |
| 25 | BLM |  |  | 811 | 0.156 | 0.5245 | Yes |
| 26 | GTSE1 |  |  | 852 | 0.152 | 0.5344 | Yes |
| 27 | MSH2 |  |  | 885 | 0.150 | 0.5449 | Yes |
| 28 | CDK1 |  |  | 915 | 0.147 | 0.5556 | Yes |
| 29 | CLSPN |  |  | 922 | 0.146 | 0.5688 | Yes |
| 30 | NDC80 |  |  | 1087 | 0.131 | 0.5624 | Yes |
| 31 | TTK |  |  | 1177 | 0.123 | 0.5639 | Yes |
| 32 | RAD51AP1 |  |  | 1196 | 0.122 | 0.5734 | Yes |
| 33 | NUSAP1 |  |  | 1338 | 0.113 | 0.5678 | Yes |
| 34 | PRIM1 |  |  | 1411 | 0.108 | 0.5697 | Yes |
| 35 | CDCA7 |  |  | 1440 | 0.106 | 0.5766 | Yes |
| 36 | ANP32E |  |  | 1548 | 0.101 | 0.5738 | No |
| 37 | MCM2 |  |  | 1762 | 0.088 | 0.5576 | No |
| 38 | AURKB |  |  | 1838 | 0.084 | 0.5570 | No |
| 39 | KIF2C |  |  | 1985 | 0.077 | 0.5474 | No |
| 40 | CBX5 |  |  | 2001 | 0.076 | 0.5529 | No |
| 41 | BRIP1 |  |  | 2104 | 0.072 | 0.5480 | No |
| 42 | UHRF1 |  |  | 2136 | 0.071 | 0.5512 | No |
| 43 | GAS2L3 |  |  | 2174 | 0.069 | 0.5535 | No |
| 44 | POLD3 |  |  | 2204 | 0.068 | 0.5566 | No |
| 45 | CDCA3 |  |  | 2232 | 0.067 | 0.5599 | No |
| 46 | MCM6 |  |  | 2237 | 0.067 | 0.5657 | No |
| 47 | CDCA8 |  |  | 2281 | 0.065 | 0.5670 | No |
| 48 | POLA1 |  |  | 2330 | 0.063 | 0.5674 | No |
| 49 | MCM5 |  |  | 2344 | 0.063 | 0.5719 | No |
| 50 | WDR76 |  |  | 2554 | 0.055 | 0.5531 | No |
| 51 | CDC45 |  |  | 2615 | 0.053 | 0.5512 | No |
| 52 | CCNE2 |  |  | 2692 | 0.050 | 0.5472 | No |
| 53 | DTL |  |  | 2697 | 0.050 | 0.5515 | No |
| 54 | RRM2 |  |  | 2835 | 0.046 | 0.5401 | No |
| 55 | RANGAP1 |  |  | 3009 | 0.041 | 0.5240 | No |
| 56 | HJURP |  |  | 3235 | 0.035 | 0.5013 | No |
| 57 | CKAP2 |  |  | 3236 | 0.035 | 0.5047 | No |
| 58 | TIPIN |  |  | 3336 | 0.032 | 0.4963 | No |
| 59 | NCAPD2 |  |  | 3371 | 0.031 | 0.4954 | No |
| 60 | FEN1 |  |  | 3406 | 0.030 | 0.4943 | No |
| 61 | CCNB2 |  |  | 3502 | 0.028 | 0.4860 | No |
| 62 | AURKA |  |  | 3503 | 0.028 | 0.4887 | No |
| 63 | PSRC1 |  |  | 3675 | 0.024 | 0.4712 | No |
| 64 | CKAP2L |  |  | 3787 | 0.020 | 0.4604 | No |
| 65 | G2E3 |  |  | 3840 | 0.019 | 0.4562 | No |
| 66 | PCNA |  |  | 3986 | 0.016 | 0.4409 | No |
| 67 | CDC6 |  |  | 4028 | 0.014 | 0.4375 | No |
| 68 | CDCA2 |  |  | 4066 | 0.013 | 0.4346 | No |
| 69 | TACC3 |  |  | 4146 | 0.011 | 0.4265 | No |
| 70 | UNG |  |  | 4979 | -0.010 | 0.3315 | No |
| 71 | RPA2 |  |  | 5078 | -0.013 | 0.3214 | No |
| 72 | UBR7 |  |  | 5204 | -0.016 | 0.3084 | No |
| 73 | HMGB2 |  |  | 5479 | -0.023 | 0.2791 | No |
| 74 | DSCC1 |  |  | 5503 | -0.024 | 0.2787 | No |
| 75 | CENPA |  |  | 5506 | -0.024 | 0.2808 | No |
| 76 | SLBP |  |  | 5661 | -0.029 | 0.2658 | No |
| 77 | CTCF |  |  | 5766 | -0.032 | 0.2568 | No |
| 78 | CDC20 |  |  | 5818 | -0.033 | 0.2541 | No |
| 79 | USP1 |  |  | 5963 | -0.038 | 0.2411 | No |
| 80 | TUBB4B |  |  | 6059 | -0.042 | 0.2341 | No |
| 81 | GMNN |  |  | 6392 | -0.052 | 0.2007 | No |
| 82 | CKS2 |  |  | 6569 | -0.059 | 0.1860 | No |
| 83 | GINS2 |  |  | 7545 | -0.122 | 0.0851 | No |
| 84 | CKS1B |  |  | 7569 | -0.124 | 0.0943 | No |
| 85 | BIRC5 |  |  | 7735 | -0.144 | 0.0890 | No |
| 86 | RFC2 |  |  | 7739 | -0.144 | 0.1024 | No |
| 87 | TYMS |  |  | 7792 | -0.150 | 0.1107 | No |
Table: GSEA details [plain text format]

  

Fig 2: SEURAT\_CELLCYCLE: Random ES distribution      
 Gene set null distribution of ES for **SEURAT\_CELLCYCLE**
